# The Impact of the Workload and Traumatic Stress on the Presenteeism of Midwives: The Mediating Effect of Psychological Detachment

**DOI:** 10.1155/2023/1686151

**Published:** 2023-06-27

**Authors:** Weiwei Jiang, Yiting Wang, Jiahua Zhang, Danni Song, Congshan Pu, Chunjian Shan

**Affiliations:** ^1^School of Nursing, Nanjing Medical University, Nanjing, China; ^2^Obstetrics Department, Women's Hospital of Nanjing Medical University (Nanjing Maternity and Child Health Care Hospital), Nanjing, China

## Abstract

**Objectives:**

Midwives are at high risk of presenteeism, which may impact the quality of midwifery and maternal and infant health outcomes. However, no research has been conducted to investigate the relationship between workload, traumatic stress, psychological detachment, and presenteeism among midwives. This study, therefore, aimed at exploring the mediating effects of psychological detachment on workload, traumatic stress, and presenteeism among midwives.

**Method:**

A multicenter cross-sectional study was conducted among 547 midwives in Jiangsu Province. Participants completed the National Aeronautics and Space Administration task load index, traumatic stress impact subscale for midwives, psychological detachment scale, and Stanford presenteeism scale and provided sociodemographic information. The mediating effects of psychological detachment were assessed using Mplus.

**Results:**

The mean score of midwives' presenteeism was 17.09±3.56. Presenteeism was positively associated with both workload and traumatic stress (both *P* < 0.01) and negatively associated with psychological detachment (*P* < 0.01) among midwives. Psychological detachment partially mediated the relationships between (a) workload and presenteeism (*β* = 0.005, *P* < 0.05) and (b) traumatic stress and presenteeism (*β* = 0.006, *P* < 0.05), with mediating effects of 11.90% and 10.00%, respectively.

**Conclusions:**

Presenteeism among midwives is at moderately high levels and requires attention from nursing managers. Psychological detachment is a mediating variable of the relationship between workload, traumatic stress, and presenteeism among midwives. *Implications for Nursing Management*. This study has implications for decreasing midwives' presenteeism in practical terms. Specifically, it is crucial that care managers attempt to adjust midwives' work patterns, reduce their workload, and provide organizational support for work-related traumatic stress. Moreover, our findings also indicate that psychological detachment is probably an essential element that is worthy of attention.

## 1. Background

Presenteeism is typically defined as an employee's perseverance to go to work when they are ill. With the evolution of its conceptualization, the definition has been extended to behaviours that result in the loss of productivity or work capability caused by physical and/or psychological (i.e., cognitive or emotional) issues [1]. In a previous study, approximately 88% of employees and 85% of healthcare workers reported that they had worked while sick [2]. It is estimated that presenteeism costs the United States economy between $150 billion and $250 billion annually [3]. In comparison with absenteeism, presenteeism is more prevalent and potentially has a more devastating impact on employee health and economic business costs [1, 3].

Evidence that the situation of presenteeism is particularly grave in the nursing profession is mounting [4, 5]. In Switzerland, for example, the presenteeism rate for nurses is 36.5% [6], in the United States, it is 78.4% [7], whilst in China, it is as high as 94.3% [8]. The chronic work overload, lack of organizational resources, work-family imbalance, high burnout rate, and lower career satisfaction [9, 10] experienced by nurses make them more susceptible to presenteeism. In addition to increasing the likelihood of poor patient prognosis such as medication errors and patient falls, nurse presenteeism has also been proven to place a financial burden on healthcare organizations worldwide [3]. However, a recent study in which a mental health survey was conducted for different healthcare professionals revealed that presenteeism was more prevalent among midwives (55.3%) compared to nurses and doctors (49.3% and 30.3%, respectively) [11]. Midwives are a vital cornerstone in reducing maternal fatalities by providing quality care to women, effectively preserving the safety of birth [12]. The quality of midwifery care is closely associated with the birth experience and maternal and infant safety, whereas the presenteeism of midwives has a crucial impact on the quality of clinical midwifery. Understanding midwives' presenteeism is a critical step in promoting increased and stable births and maximizing midwifery productivity and the scope of practice. There is, however, a dearth of research on presenteeism in the midwifery profession, both in China and in other countries. Therefore, it is important to investigate the factors influencing the presenteeism of midwives in China to maintain a healthy and motivated midwifery workforce.

A key predictor of nurses' presenteeism is work-related stress. Currently, the world's population is expanding rapidly, while the number of midwives is substantially declining [13]. In comparison with the WHO standard of six midwives per 1,000 births, the ratio of midwives to the number of women in labour in China is only one in four thousand, revealing a severe workforce shortage for midwifery [14]. High workloads and time constraints brought on by a lack of human resources demonstrate the high level of quantifiable job demands that midwives need to undertake [15], while frequent invasive operations and shift work further aggravate the mental workload of midwives [13]. Previous studies showed that an overloaded work status played an important role in the occurrence of presenteeism among employees [16, 17]. When employees are exposed to excessive workloads, work-family imbalance and low occupational well-being may lead to role overload and internal role conflicts, which subsequently affect employees' physical and mental health (e.g., occupational fatigue, emotional burnout, and presenteeism). Therefore, we propose Hypothesis 1: workload positively affects presenteeism among midwives.

In addition, delivery room as a special medical unit, maternal or foetal death, neonatal resuscitation, postpartum haemorrhage, and other traumatic workplace events have become major causes of job stress for midwives [18, 19]. Traumatic stress in midwives usually refers to the psychological stress response to an adverse birth event directly experienced or witnessed in the workplace [20, 21]. Traumatic stress symptoms, in contrast to secondary traumatic stress (STS), are less severe and may be an early warning indicator of psychiatric disorders such as STS [18, 21, 22]. A systematic review [21] indicated that approximately 71%–96.9% of midwives had directly or indirectly experienced a traumatic birth event while working, and such experiences had varying degrees of negative consequences on the physical and mental health of midwives, with 12.6%–38.7% presenting with symptoms of STS and 35%–66% presenting with subclinical symptoms of posttraumatic stress disorder (PTSD). Dunbar et al. [23] reported that US military personnel who experienced anxiety, depression, and PTSD after a traumatic event had a significantly higher incidence of presenteeism. Similarly, after experiencing or witnessing traumatic events, midwives often experience negative feelings, including sadness, dreadfulness, empathic fatigue, and even physical symptoms such as insomnia and weight loss [24, 25]. The negative outcomes contradict the public perception of childbirth, and the risk-filled work atmosphere often leads to exhaustion, preventing midwives from realizing the full potential and effectiveness of the art of childbirth care. Therefore, we propose Hypothesis 2: traumatic stress positively affects presenteeism among midwives.

As the effects of work stress on mental health and behaviour at work have received attention, the experience of recovery has been recognized as one of the crucial mechanisms that may constitute these interactions. Previous studies have shown that the experience of recovery is inversely correlated with presenteeism [16, 17]. Psychological detachment, as a core experience of work recovery, means that individuals are physically and mentally detached from jobs during nonworking hours [26, 27], which has an equally important impact on employees' presenteeism. Specifically, Huyghebaert-Zouaghi et al. [28] reported that employees' psychological detachment was negatively associated with presenteeism. Employees' mental and physical functions are constantly activated when they are frequently exposed to high work demands [29, 30]. Prolonged low relaxation experiences and high emotional burnout can lead to negative outcomes (e.g., sleep disorders, low work engagement, and presenteeism) if there is no complete detachment from work to bring physical and mental stress back to baseline levels [31].

**Figure 1:**
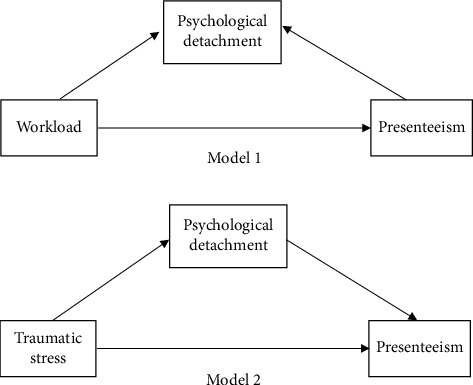
Conceptual framework.

However, this critical recovery process can also be compromised by work-related traumatic stress. According to the effort-recovery theory, continuing psychological activation (such as prolonged exposure to intense work demands like overtime and shift work) and cognitively related stress processes (e.g., rumination) are two situations that can impede the recovery process [31]. Newman et al. [32] noted that prolonged direct or indirect experiences of traumatic events in the workplace can potentially contribute to increased mental distress (e.g., anxiety, distress, and depression) and physical symptoms (e.g., insomnia and panic disorder) in forensic mental health nurses, which in turn influence their work efficacy. Multiple qualitative studies [33–35] have similarly noted that, in response to immutable distressing birth outcomes, midwives may suffer self-doubt, rumination, and professional alienation, while repeated flashbacks to traumatic scenarios keep them from separating from the traumatic event. As mentioned above, psychological detachment can be considered a mediator to explain the potential mechanisms of how exposure to high workloads and traumatic stress affects presenteeism among midwives. However, although such potential mediating mechanisms may exist, few studies have explored them. Therefore, we propose the last two hypotheses: psychological detachment mediates the relationship between workload and presenteeism among midwives (Hypothesis 3), and psychological detachment mediates the relationship between traumatic stress and midwives' presenteeism (Hypothesis 4). The hypotheses of Model 1 and Model 2 for this study are shown in Figure 1.

## 2. Methods

### 2.1. Study Design and Participants

A multicenter, cross-sectional survey was conducted in Jiangsu Province, China. From August 2022 to September 2022, a convenience sample of 32 hospitals in Northern Jiangsu (Xuzhou, Suqian, and Huai'an), Central Jiangsu (Nantong and Yangzhou), and Southern Jiangsu (Nanjing, Suzhou, and Wuxi) was used. These hospitals were selected because they are long-term partners in our research project and they provide midwifery services with a high level of medicine, which is useful for investigating presenteeism among midwives in Jiangsu. Finally, eligible midwives from the selected hospitals were conveniently selected for inclusion. Midwives with a Chinese Practicing Nurse certificate, a Certificate of Maternal and Child Health Care, and a minimum of three months of experience as a midwife were selected for the study. Those who were not present during the survey period (due to long-term leave or additional training, for example) were excluded. According to Kendall's principle of sample estimation, the sample size was 5–10 times the number of variables, and 40 variables were included in this study. Considering an invalid response rate of 10%, the sample size was equal to 220∼440 in the current study. A total of 587 questionnaires were collected, and after excluding 40 invalid questionnaires (e.g., with options that all provided the same score), 547 valid questionnaires were included in the final analysis (93.2% validity rate). To ensure adequate and complete reporting of this research, we used the Strengthening the Reporting of Observational Studies in Epidemiology (STROBE) guideline [36] for cross-sectional studies (File S1).

### 2.2. Measurements

#### 2.2.1. Participants' General Characteristics

Ten questions on sociodemographic and professional features were included in the self-designed questionnaire, including gender, age, years of work, education level, monthly income, hospital level, marital status, number of children, number of overtime hours, and professional title.

#### 2.2.2. National Aeronautics and Space Administration Task Load Index (NASA-TLX)

The Chinese version of the NASA-TLX scale was adapted by Liang et al. [37] and originally developed by Hart and Staveland [38] to measure the mental workload of nurses. It was divided into two dimensions, load perception and self-evaluation, which contained the following six items: mental demand, physical demand, temporal demand, performance, effort, and frustration. Each item was represented by a straight line that had been divided into 20 equal scores ranging from 0 to 20, indicating low to heavy load. The scores of all items were totalled to determine the overall score, and a higher total score indicated a higher workload for a nurse. Cronbach's alpha coefficient of the Chinese version of the NASA-TLX was 0.714. In this study, Cronbach's alpha coefficient for this scale was 0.792.

#### 2.2.3. Traumatic Stress Impact Subscale for Midwives

The Traumatic Stress Scale for Midwives was developed by Kubota and Horiuchi [22]. After obtaining authorization from the authors of the original scale, this research team first translated the scale into Chinese according to the Brislin translation model and evaluated the reliability and validity of the scale by surveying 385 midwives. The Chinese version of the Traumatic Stress Scale for Midwives included 15 items, divided into two subscales (the frequency of occurrence and degree of impact subscales), and Cronbach's alpha coefficient of the Chinese version of the scale was 0.888∼0.953, the retest reliability was 0.824∼0.884, and the content validity index was 0.700. In this study, the Traumatic Stress Impact Subscale for Midwives was measured using a 4-point Likert scale, with each item scored from 0 (completely unaffected) to 3 (very affected) and a total score of 0 to 45, with higher scores representing a higher degree of impact of a traumatic stress event on a midwife. Cronbach's alpha for this subscale was 0.963.

#### 2.2.4. Psychological Detachment from Work

The Chinese version of the psychological detachment scale was adapted by Lu et al. [39] and originally developed by Sonnentag and Fritz [29] to measure the state in which employees were mentally isolated from their work and avoided thinking about work-related issues. This scale included four items, such as “I distance myself from my work.” A 5-point Likert scale ranging from 1 (strongly disagree) to 5 (strongly agree) was used to indicate the level of psychological detachment in midwives. Lower scores indicated that midwives were less able to disconnect well from work. Cronbach's alpha coefficient for the Chinese version of this scale was 0.844. Cronbach's alpha for this scale in this study was 0.852.

#### 2.2.5. Presenteeism

The Chinese version of the Stanford Presenteeism Scale was adapted by Zhao et al. [40] and originally developed by Koopman et al. [41] to assess presenteeism and estimate health-related productivity loss. This scale consisted of six items with two dimensions as follows: finishing work (four items) and avoiding distractions (two items). All items were rated on a 5-point Likert scale ranging from 1 (strongly disagree) to 5 (strongly agree). Higher scores indicated higher presenteeism or significant productivity loss. Cronbach's alpha coefficient for the Chinese version of this scale was 0.862. In this study, Cronbach's alpha coefficient for this scale was 0.765.

### 2.3. Ethical Considerations

The study was approved by the Ethics Committee of Nanjing Maternity and Child Health Care Hospital (2022KY-090-01). All participants provided informed consent for this study.

### 2.4. Data Collection

All data were collected through the online questionnaire platform “Wenjuanxing.” With the permission of the person in charge of the surveyed hospital, the nurse managers of each hospital delivery room were set up as investigators and trained online by the research team. The trained nurse managers informed midwives of the purpose, significance, and content of the study. When a midwife was willing to participate, the nurse manager verified that they met the inclusion criteria and then sent a link to the questionnaire separately via the WeChat app. The informed consent form and instructions for completion appeared on the first page of the electronic questionnaire again, and consent was deemed to have been obtained if participants clicked on the link and completed the survey. The study was anonymous and could only be submitted successfully if all options were completed. A survey setting option that allowed only one response per participant was selected to avoid duplicate responses.

### 2.5. Data Analysis

Statistical analyses were conducted using IBM SPSS Statistics 26.0 software. Participant demographic characteristics were analysed using descriptive statistics, including frequencies, percentages, and means ± SDs, and independent *t* tests, one-way ANOVA tests, and nonparametric tests were applied to determine the differences in midwives' presenteeism under varying demographic characteristics. Based on a two-tailed test, a result of *P* < 0.05 was deemed statistically significant. Workload, traumatic stress, psychological detachment, and presenteeism among midwives were tested for bivariate association coefficients using Pearson correlation coefficients. The mediating effects were analysed using Mplus version 7.0. In this study, workload and traumatic stress were posited as independent variables, presenteeism was posited as the dependent variable, psychological detachment was posited as the mediating variable, and statistically significant sociodemographic variables were set as covariates. With 5,000 bootstrapping resamples, a 95% confidence interval (CI) was obtained. The indirect impact was considered significant if the 95% CI for the mediation path did not contain zero. The following criteria were used to appraise the model fit: *χ*^2^/DF ≤ 5.00, comparative fit index (CFI) ≥0.90, Tucker–Lewis index (TLI) ≥ 0.90, standardized root mean square residual (SRMR) ≤ 0.08, and root mean square error of approximation (RMSEA) ≤ 0.08 [42].

## 3. Results

### 3.1. Characteristics of Midwives

A total of 547 midwives were included in the analytical sample listed in Table 1. Among the participants, all of whom were female, 76.78% were under the age of forty, and more than half (63.80%) had been working as midwives for over ten years. The majority of them (90.31%) held a bachelor's degree or above. Moreover, 76.42% of the respondents were married, and 70.93% had at least one child. The participants' demographic details are displayed in Table 1.

### 3.2. Single-Variable Analysis of Presenteeism with Different Demographic Variables

The differences in midwives' presenteeism according to demographic characteristics are shown in Table 1. There were significant differences in presenteeism among midwives depending on age (*F* = 4.606, *P*=0.003), monthly income (*H* = 7.277, *P* = 0.026), and hospital level (*F* = 5.412, *P* = 0.001), but not in other factors such as years of work, education level, marital status, or professional title.

### 3.3. Descriptions and Correlations of Variables

Table 2 displays the descriptive statistics and correlations of workload, traumatic stress, psychological detachment, and presenteeism among midwives. The average presenteeism score was 17.09 ± 3.56, with a score range of 6 to 30, which indicated a medium to high level. High workload (85.45 ± 18.20) and traumatic stress levels (21.90 ± 11.69) as well as somewhat moderately low levels of psychological detachment (9.51 ± 3.61) were perceived by the midwives. In addition, Pearson's correction analysis showed that workload and traumatic stress were negatively correlated with psychological detachment and positively correlated with presenteeism among midwives, respectively, while psychological detachment was negatively correlated with presenteeism, as shown in Table 2.

### 3.4. Testing the Hypothesized Model

**Figure 2:**
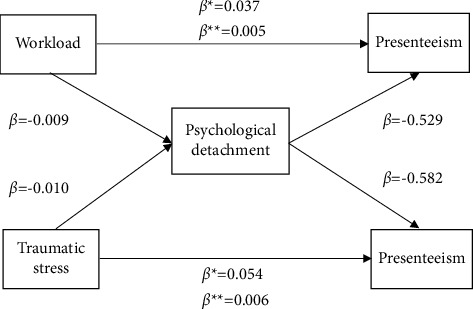
Mediating effects of psychological detachment on the association between workload, traumatic stress, and presenteeism. Note: *β*^*∗*^ = direct effect and *β*^*∗∗*^ = indirect effect.

After controlling for age, monthly income, and hospital level, the model fits for both Model 1 and Model 2 were acceptable (*χ*^2^/DF = 1.110, CFI = 0.997, TLI = 0.991, RMSEA = 0.014, and SRMR = 0.024 and *χ*^2^/DF = 1.206, CFI = 0.998, TLI = 0.995, RMSEA = 0.010, and SRMR = 0.019), respectively. The findings of the hypothesis test after controlling for covariates are listed in Table 3. The total effects of workload and traumatic stress on presenteeism were significant (*β* = 0.042 and 0.060, respectively, *ps* < 0.001). Moreover, psychological detachment accounted for 11.90% and 10.00% of the combined effects of workload and traumatic stress on presenteeism, serving as a partial mediator of the relationship between workload, traumatic stress, and presenteeism. Since neither CI contained zero, we deduced that psychological detachment had a significant impact on the link between presenteeism, traumatic stress, and mental workload. The mediating role of psychological detachment in the relationship between workload, traumatic stress, and presenteeism is shown in Figure 2.

## 4. Discussion

Given the distinct occupational nature of midwifery and in contrast to the previous studies that focused on nurses' presenteeism, this study also took into account the close relationship between occupational stress caused by traumatic events during childbirth and midwives' presenteeism. To the best of our knowledge, this study, which links workload, traumatic stress, psychological detachment, and presenteeism for the first time, sheds important light on the working behaviours of Chinese midwives and how they relate to stressful psychological work characteristics and recovery experiences.

Midwives who participated in this study reported a moderately high level of presenteeism, which was higher than the results of other research on presenteeism among Chinese nurses [43, 44], suggesting that the phenomenon of midwives' presenteeism is relatively serious in the Chinese nursing population. The possible reasons are as follows: (1) midwives attend to vulnerable groups such as expectant mothers and newborns, which calls for more humanistic care and emotional commitment and are prone to behavioural and psychological overload, leading to fatigue and presenteeism behaviours [45]. (2) Given that the midwifery system in China is still developing, the functional boundaries between midwives and obstetric nurses remain unclear, and midwives continue to perform a lot of basic tasks while providing specialized services [46]. Professional fatigue and emotional burnout lead to role overload, which prevents them from being fully engaged in their work. (3) Teams of midwives include members with distinct roles and duties. Absence will increase the workload of other members and affect teamwork; owing to the sense of teamwork and responsibility, the majority of midwives opt for presenteeism [4]. Therefore, Chinese nursing managers should pay attention to the phenomenon of midwives' presenteeism and be concerned about their work experiences and health status. Meanwhile, providing organizational support and a harmonious working environment safeguards a good physical and mental status and reduces midwives' presenteeism.

Based on the results of the mediation model, workload and traumatic stress directly and positively influenced midwives' presenteeism, supporting Hypothesis 1 and Hypothesis 2. Similar results were obtained by Baek et al. [16], who found that workload was positively associated with presenteeism. The workload of the midwives in this study was at a moderately high level, meaning they were under heavy psychological work stress. Cull et al. [15] reported that the increasing number of nonclinical roles such as emotional effort, physical effort, and professional skill requirements placed a heavy burden on midwives and gradually diminished their motivation for their jobs, resulting in burnout and decreased productivity. Moreover, the COVID-19 pandemic exacerbated the shortage of midwives, which further added to their work burden. Trying to cope with the changes and challenges of a pandemic along with the fear of infection increased midwives' burnout and diminished job satisfaction and security [47, 48]. According to the job demand-control (JDC) model, employees in high-demand jobs are inclined to exert more physical and psychological effort to maintain a high level of performance [49]. These common load reactions can develop into more severe chronic load reactions over time, which worsen job fatigue and may increase presenteeism. In addition, presenteeism is also attributed to the traumatic stress of midwives, and the more traumatic stress they experience, the more likely presenteeism is to occur. This result is consistent with a previous study of US military personnel [23]. A mixed-methods study [35] reported that, as secondary victims, midwives were vulnerable to physical and emotional pain such as sadness, guilt, self-blame, and empathic fatigue after witnessing or experiencing traumatic events. Owing to their professional role, the midwives had to keep working, but their inability to devote themselves to their work caused a decrease in their productivity.

The more important finding of this study was that psychological detachment partially mediated the role of workload and traumatic stress in relation to midwives' presenteeism, which confirmed Hypotheses 3 and 4. This means that when midwives are subjected to high workloads and traumatic stress, their level of psychological detachment is reduced, which in turn increases presenteeism. As reported in the studies [15, 35, 50], midwives were under heavy workloads and inevitably feared that their work mistakes would endanger the health of mothers and children even after birth, which made it impossible for them to truly detach from their work. Additionally, after experiencing traumatic events, midwives might suspect or even lose confidence in their midwifery skills and career, resulting in fear of childbirth and midwifery, as well as fear that their care decisions might negatively affect mothers and babies or even aggravate the traumatic event, which caused their psychological burden to increase and hindered their psychological detachment [35, 50]. According to the effort-recovery model, a high level of psychological detachment, as a positive emotional-motivational state, and resilience allows nurses to recover experiences and emotional resources that have been depleted in the workplace, which is an important ability to improve patient safety and work focus [51]. When midwives have higher levels of psychological detachment, they are in a better position to adjust themselves in the face of high workload and traumatic stress levels, which in turn supports reduced negative emotions at work, increased job engagement and professional confidence, and lessened presenteeism [17, 18]. This result suggests that nursing managers can reduce midwives' presenteeism not only by relieving their workload and providing support programs such as counselling and stress management training to moderate their traumatic stress levels but also by implementing different interventions to improve their psychological detachment to improve performance and in turn reduce presenteeism.

## 5. Limitations

When interpreting these results, it should be considered that the study has several limitations. First, as the sampling method used in this study was convenience sampling rather than stratified sampling, a relatively large sampling error could not be avoided. Second, we only collected data from Jiangsu Province, China, to test our hypothesis, which may be geographically limited and cannot be widely generalized. Thus, the regional sample should be expanded in the future to improve the universality of the study results. Third, this study used a cross-sectional design, which may bias the results and fail to provide a specific causal relationship between workload, traumatic stress, and presenteeism. It would be worthwhile to validate this model using a longitudinal study to see if the negative outcomes of high workload and traumatic stress increase over time. Finally, this study only considered psychological detachment as a potential mechanism between workload, traumatic stress, and presenteeism, while other factors such as empathic fatigue, emotional exhaustion, and work alienation could be considered as mediators in future studies. Despite these limitations, we report meaningful information on midwives' workloads, traumatic stress, psychological detachment, and the correlation with presenteeism.

## 6. Conclusion

The results showed that presenteeism among midwives was serious. Workload and traumatic stress can affect midwives' presenteeism either via direct pathways or through mediators of psychological detachment. Assessing and relieving midwives' workloads and traumatic stress may therefore be crucial in lessening midwives' presenteeism and improving their psychological detachment. Stress management and work recovery experience-oriented interventions ought to be provided to midwives by nurse managers to help them cope positively with frustration or stress and to detach and recover from high work stress. Further research should continue to explore the modifiable risks and protective factors associated with midwives' presenteeism to enhance their work engagement.

## 7. Relevance to Clinical Practice

This study provides several insights for nurse managers. First, nurse managers should be fully aware of the key role of midwives in maternal and child health services, further strengthen the midwifery system, and stratify the job functions of senior midwives and obstetric nurses to reduce the workload of midwives, which is essential for reducing the incidence of presenteeism among midwives. Second, teaching methods such as role-playing and scenario-based simulation are effective in enhancing midwives' decision-making and clinical management skills in labour [52]. These measures serve to encourage midwives to rebuild their cognition of traumatic events and develop positive coping mechanisms and to lessen the mental and physical impact of traumatic events on midwives, which in turn reduces the incidence of presenteeism [52]. Finally, nurse managers could implement practical strategies such as mindfulness, boundary management, and sleep cognitive therapy to reduce rumination on work-related stress and improve mental detachment among midwives to facilitate work recovery experiences, which ultimately reduces presenteeism [53].

## Figures and Tables

**Table 1:** Demographic characteristics of different participants and univariate analysis of presenteeism (*n* = 547).

Variable	Group	*n*	%	Score (mean ±SD)	Test value	*P*
Years of work	≤3	38	6.95	18.05±3.73	1.965^†^	0.098
	4∼6	66	12.07	17.06±3.08		
	7∼9	94	17.18	16.38±3.66		
	10∼14	173	31.62	17.38±3.65		
	≥15	176	32.18	13.52±3.52		

Age (years)	18∼29	171	31.26	16.26±3.04	4.606^†^	0.003
	30∼39	249	45.52	17.47±3.68		
	40∼49	100	18.28	17.52±3.74		
	≥50	27	4.94	17.15±4.12		

Education level	Technical secondary school	7	1.28	15.86±2.41	0.320^†^	0.811
	Junior college	46	8.41	17.13±2.87		
	Bachelor's degree	490	89.58	17.11±3.65		
	Master's degree or above	4	0.73	16.50±1.91		

Monthly income (RMB)	<5000	93	17.00	18.02±4.14	7.277^‡^	0.026
	5000∼10000	375	68.56	17.02±3.47		
	>10000	79	14.44	16.33±3.05		

Hospital level	Level A tertiary hospital	251	45.88	17.39±3.48	5.412^†^	0.001
	Level B tertiary hospital	162	29.62	17.41±3.89		
	Level A secondary hospital	114	20.84	16.33±2.92		
	Level B secondary hospital	20	3.66	14.90±4.10		

Marital status	Single	115	21.02	16.72±3.20	0.768^†^	0.464
	Married	418	76.42	17.19±3.65		
	Widowed or separated	14	2.56	17.14±3.76		

Number of children	0	159	29.07	16.77±3.17	2.633^†^	0.073
	1	293	53.56	17.02±3.59		
	≥2	95	17.37	17.81±4.03		

Overtime hours (weekly)	>5	172	31.44	17.27±3.59	0.824^§^	0.410
	≤5	375	68.56	17.00±3.56		

Professional title	Junior	247	45.16	16.92±3.33	0.862^†^	0.423
	Intermediate	213	38.94	17.34±3.57		
	Senior	87	15.90	16.95±4.17		

*Note*. ^†^indicates a one-way ANOVA test. ^§^indicates an independent *t* tests. ^‡^indicates a Kruskal–Wallis H test.

**Table 2:** Variables' descriptive statistics and correlation analysis (*n* = 547).

Variable	Score range	Mean	SD	1	2	3	4
(1) Workload	0∼120	85.45	18.20	1			
(2) Traumatic stress	0∼45	21.90	11.69	0.139^*∗∗*^	1		
(3) Psychological detachment	4∼20	9.51	3.61	−0.190^*∗∗*^	−0.126^*∗∗*^	1	
(4) Presenteeism	6∼30	17.09	3.56	0.249^*∗∗*^	0.218^*∗∗*^	−0.188^*∗∗*^	1

SD: standard deviation. ^*∗∗*^*P* < 0.01.

**Table 3:** Estimates of the hypothesized model's effects (*n* = 547).

Structural paths	Effect	SE	*t*	*P*	95% CI	
					LLCI	ULCI
Total effects						
Workload ⟶ presenteeism	0.042	0.008	5.086	<0.001	0.026	0.058
Traumatic stress ⟶ presenteeism	0.060	0.013	4.802	<0.001	0.036	0.085
Direct effects						
Workload ⟶ psychological detachment	−0.009	0.002	−4.190	<0.001	−0.014	−0.005
Psychological detachment ⟶ presenteeism	−0.529	0.164	−3.231	0.001	−0.835	−0.199
Workload ⟶ presenteeism	0.037	0.008	4.554	<0.001	0.021	0.053
Traumatic stress ⟶ psychological detachment	−0.010	0.004	−2.762	0.006	−0.017	−0.003
Psychological detachment ⟶ presenteeism	−0.582	0.163	−3.584	<0.001	−0.885	−0.259
Traumatic stress ⟶ presenteeism	0.054	0.013	4.340	<0.001	0.031	0.080
Indirect effects						
Workload ⟶ psychological detachment ⟶ presenteeism	0.005	0.002^a^	2.498	0.012	0.002	0.010
Traumatic stress ⟶ psychological detachment ⟶ presenteeism	0.006	0.003^a^	2.079	0.038	0.002	0.012
Proportion mediated (workload ⟶ psychological detachment ⟶ presenteeism)^*∗*^	11.90%					
Proportion mediated (traumatic stress ⟶ psychological detachment ⟶ presenteeism)^*∗*^	10.00%					

^a^Bootstrapped standard error; ^*∗*^proportional mediation is the ratio of the indirect effect to the total effect, e.g., indirect effect/total effect 100%. CI, confidence interval; LLCI, lower limit confidence interval; SE, standard error; ULCL, upper limit confidence limit interval.

## Data Availability

The data that support the findings of this study are available on request from the corresponding author. The data are not publicly available due to privacy or ethical restrictions.
